# From the stillness of feigning death to near-death experience?

**DOI:** 10.1093/braincomms/fcab138

**Published:** 2021-06-22

**Authors:** Kevin R Nelson

**Affiliations:** University of Kentucky, Lexington, KY 40536, USA

**This scientific commentary refers to ‘The evolutionary origin of near-death experiences: a systematic investigation’, by Peinkhofer et al. (https://doi.org/10.1093/braincomms/fcab132).**

In the Gifford Lectures of 1901–02 at the University of Edinburgh, William James a physician, psychologist and philosopher categorized spiritual experiences thereby establishing the origins of their scientific study. Among the principles subsequently published in ‘The Varieties of Religious Experience’ (VRE), the mystical sense of unity or oneness dominated James’ thinking as ‘the root and center’ of spiritual experience, which he believed formed the bedrock of many organized religions. Near-death experiences receive mention in VRE, because near-death fulfils James’ criteria for a spiritual experience whereby ‘feelings, acts and experiences’ touch ‘whatever they may consider the divine’. 

For James mystical experiences eclipse the influence of near-death. However, since Raymond Moody’s ‘Life after Life’ in 1975, near-death experiences have gained wide attention, forging a deeply imbedded image of what spiritual experience means to many people today. The drama of going through a tunnel, being enveloped by ‘the light’, hovering above one’s body during crisis and meeting deceased loved ones or spiritual beings constitutes a powerful narrative repeatedly portrayed in popular media. Broad interest in near-death could also be fuelled by the likelihood of its common occurrence. Syncope in a safe laboratory environment provokes the core experiences of near-death,^1^ and syncope happens within the lifetime of upwards to a quarter of the general population.

Understanding brain mechanisms during spiritual experience has obviously advanced sharply since James’ time. Mystical experience currently enjoys a scientific and medical renaissance aided by recognizing the role of serotonergic 2a receptor agonists. Furthermore, accumulating evidence supports the notion that rapid eye movement (REM) consciousness intruding into wakefulness contributes essential elements to near-death experience. Such insights allow a fuller exploration of these sublime experiences.

In this issue, Peinkhofer and colleagues^2^ offer the hypothesis that in the course of evolution near-death experiences arose from mechanisms promoting survival through thanatosis, the behaviour of feigning death. This is an intriguing idea worth investigating. Atonia during awareness is a thread running through many near-death narratives. Nearly all near-death experiences happened when the person is lying, not upright sitting, standing, walking or running.

Their first line of evidence stems from the array of creatures showing immobility with threat. From insect to humankind, thanatosis represents convergent evolution given the diversity of nervous systems sub-serving the phenomena across phyla. Ascribing biologic purpose invites anthropomorphic bias, a caution acknowledged by the authors, for atonia and stillness bolster many survival strategies. The immobility of insects can represent threat assessment. Stillness also supports camouflage and not feigning death. Rabbits freeze, immobile but not atonic, to improve the odds of being undetected by predators. In rats, freezing signals danger to others. Prey movement triggers predator attacks, such as the North American cougar, prompting wildlife experts to advise against running when humans confront the cat. Felines carry their young to safety by lifting them by the scruff of their necks reflexively inducing atonia.

The investigators examined near-death experiences during encounters with ‘modern’ predators. This analysis is notable because the content of near-death is shaped by immediate surroundings and events as well as prior life experiences. The number of subjects with near-death provoked by ‘modern’ predators represents a clear minority of their cases. This finding seems counter to their argument, until considering how the contemporary causes of near-death are tied to predation. Many studies report the most frequent proximate cause of near-death involves a threat to cerebral blood flow (syncope or cardiac arrest), conditions readily envisioned arising from struggles with predators. Furthermore, simply believing one faces death is sufficient for experiences nearly indistinguishable from near-death in life-threatening circumstances.^3^ So nearly indistinguishable that the term ‘near-death’ borders on misnomer since in half the instances of near-death, individuals are not in medical danger.^3^ These observations remain consistent with the thanatosis hypothesis.

The principal strength of the hypothesis ties the evolutionary value of stillness to proposed mechanisms of near-death experience. In particular, REM intrusion of which atonia, useful for feigning death, constitutes a major feature. Other features of REM intruding into wakefulness include hallucinations and autoscopy. Individuals experiencing near-death have arousal systems predisposed to REM intrusion.^4^^,^^5^ To promote survival, REM mechanisms must integrate with brainstem structures and physiology critical to fight or flight and responses to cerebral ischaemia.^6^ The appropriate conscious state of wakefulness must be assured to successfully engage fight or flight. Crisis is an inopportune time to fall asleep or is it?

REM mechanisms have a long-recognized association with cardiopulmonary function. Stimulating cardiopulmonary afferents rapidly, ‘reflexively’ triggers REM in animals^7^ and strongly facilitates human REM. In Guillain–Barré syndrome the autoimmune attack on cardiac, vascular and respiratory peripheral autonomic fibres leads to florid intrusions of REM consciousness.^8^ Vagal afferents synapse within the medullary nucleus tractus solitarius, with rostral projections to the pontine parabrachial nuclear complex The parabrachial nuclear complex acts as the paramount relay for ascending cardiorespiratory afferents to the forebrain. The nucleus tractus solitarius and parabrachial nuclear complex both reciprocally connect with cholinergic REM structures. The parabrachial nuclear complex region forms an intersection where neurons functioning specifically during REM consciousness intermingle with neurons participating in the cardiorespiratory function. Although these correlations link REM consciousness and cardiopulmonary systems, understanding their relationship is incomplete.

A component of the pontine REM flip-flop switch is the ventrolateral portion of the periaqueductal grey (vlPAG). The vlPAG favours waking consciousness, functioning as a REM-off component suppressing REM. The vlPAG reacts vigorously to physiologic crisis. Pain, hypoxia and moderate blood loss stimulate the vlPAG, promoting wakefulness. Yet the vlPAG response changes when systemic blood flow becomes profoundly low. With moderate blood loss, the vlPAG supports the adrenergic system to maintain blood pressure. Paradoxically, with marked blood loss, vlPAG neurons diminish the peripheral adrenergic cardiovascular tone, bringing the cholinergic system to dominance,^9^ further aggravating systemic hypotension. As the vlPAG dampens the adrenergic nervous system, the once agitated animal with shifting attention becomes quiet and inattentive, disengaging from its surroundings. It is unknown if vlPAG neurons responsible for the response to severe hypotension bear a relationship to vlPAG REM-off neurons. Nonetheless, the vlPAG provides a potential means of triggering REM and atonia during cerebral ischaemia.

Atonia in the setting of severe hypotension could aid prey to lie quietly hidden without thrashing or issuing cries of distress, behaviour irrelevant to feigning death. Alternatively, although submission to predators following severe injury is disadvantageous to the individual, this trait could be advantageous to the species by curtailing attack upon on others. Sacrificing one for the many contains evolutionary value. Behaviour resulting from atonia can foster more than a single evolutionary advantage, with thanatosis as one. Syncopal atonia with physical collapse improving venous return to the heart, as another.

The investigators point out that thanatosis may share characteristics of laughter from tickling as a preserved behaviour with no clearly defined biologic purpose. Laughter is a well-recognized precipitant of cataplexy in orexin deficient narcolepsy, a disorder clinically defined by pathologic REM intrusion. Laughter suppresses motor neurons, possibly sharing the physiology of REM cataplexy.^10^ This point highlights that atonia comes under varied circumstances. It is more likely that a given system of atonia supports more than a single purpose rather than each atonic expression reflect a unique mechanism. The REM system generates atonia through the glutamatergic neurons of the subcoeruleus nucleus that synapse upon glycinergic/GABAergic interneurons inhibiting spinal motor neurons. This pathway stands poised to express itself in physiologic contexts like thanatosis, and beyond the weakness that sweeps over us each night in REM sleep.

Those motionless moments evoked by REM mechanisms may be one form of stillness leading to experiences presently regarded as near-death. Some find this thought unsettling, especially when reducing these experiences to vestigial consequences of primal biology. Here James offers counsel to persons whose near-death experience steadfastly transformed personal meaning and spirituality: ‘by their fruit ye shall know them, not by their roots’.
